# Autologous Platelet-Rich Plasma Eye Drops for the Treatment of Post-LASIK Chronic Ocular Surface Syndrome

**DOI:** 10.1155/2017/2457620

**Published:** 2017-12-12

**Authors:** Jorge L. Alio, Alejandra E. Rodriguez, Ahmed A. Abdelghany, Renan F. Oliveira

**Affiliations:** ^1^Division of Ophthalmology, School of Medicine, Universidad Miguel Hernández, Alicante, Spain; ^2^Refractive Surgery Department, VISSUM, Alicante, Spain; ^3^Research & Development Department and Cornea and Ocular Surface Department, VISSUM, Alicante, Spain; ^4^Division of Ophthalmology, Universidad Miguel Hernández, Alicante, Spain; ^5^Ophthalmology Department, Faculty of Medicine, Minya University, Minya, Egypt; ^6^Research & Development Department, VISSUM, Alicante, Spain

## Abstract

**Purpose:**

To evaluate the efficacy of autologous platelet-rich plasma (E-PRP) eye drops for the treatment of chronic ocular surface syndrome (OSS) following laser in situ keratomileusis (LASIK).

**Methods:**

This prospective interventional consecutive clinical study include 156 eyes of 80 patients affected by post-LASIK chronic OSS who were treated with autologous E-PRP 6 times a day as monotherapy for 6 weeks.

**Results:**

Dry eye symptoms improved in 85% of the cases. A decrease in at least one quadrant to total disappearance on CFS was observed in 89.6% of the patients who had positive CFS before treatment. Three eyes presented severe punctate keratitis (1.9%) at baseline, all of which healed completely. Conjunctival hyperemia improved in 93.3% of the patients with previous signs of ocular surface inflammation. There was a significant improvement in logMAR CDVA from 0.14 ± 0.19 to 0.06 ± 0.12 (*p* = 0.000), and 74 (71.4%) eyes improved at least 1 line in CDVA.

**Conclusion:**

Monotherapy with autologous E-PRP is a well-tolerated, safe, and effective treatment for the management of post-LASIK ocular surface syndrome.

**Precis:**

Monotherapy with autologous platelet-rich plasma eye drops has been shown to be an adequate option for the treatment of post-LASIK chronic ocular surface syndrome. This trial is registered with NCT03322917.

## 1. Introduction

Laser in situ keratomileusis (LASIK) surgery is the most commonly performed refractive surgical procedure. Postoperative complications are rare, and patient satisfaction is generally excellent, with 95.4% of the patients indicating satisfaction when a survey is conducted 7 or more months after LASIK [1].

Although usually transitory, signs and symptoms of tear dysfunction after LASIK occur in nearly all patients, with spontaneous resolution in the vast majority of cases. However, some patients may develop chronic ocular surface dysfunction that lasts for 6 months or longer. Post-LASIK ocular surface syndrome (OSS) is a term used to describe a spectrum of dry eye disease (DED) characterized by symptoms of dry eye, persistent neurotrophic epitheliopathy, tear film instability, true aqueous tear deficiency, decreased corrected distance visual acuity (CDVA) and visual quality, and neuropathic pain states [2–13]. This clinical condition has also received different names, being the term LASIK-induced neurotrophic epitheliopathy (LINE) as one of the most frequently used [4, 5]. Signs or symptoms of DED after LASIK have been found to be present in 50% of the patients at 1 week postoperatively, 40% at 1 month, and 20–40% at 6 months [6].

These changes in the ocular surface are caused by multiple factors inherent to the LASIK technique. The transection of large numbers of afferent sensory nerve fibers during the lamellar cut results in early postoperative corneal hypoesthesia, which decreases neurotrophic influence on epithelial cells and disrupts the ocular surface-lacrimal gland functional unit, leading to decreased reflex and basal tear production as well as blinking rate, with consequent tear film hyperosmolarity and ocular surface inflammation [2, 4, 5, 10, 11]. Additional causes for post-LASIK OSS include a reduction in goblet cell density and mucin production, leading to tear film instability [12, 13], and altered tear film distribution due to changes in the corneal shape [2, 4, 5].

Most cases of early postoperative dry eye symptoms were resolved with appropriate management, which may include nonpreserved lubricant eye drops, punctal plugs, soft contact lenses, oral supplementation with omega-3 essential fatty acids, and/or topical anti-inflammatory agents such as 0.05% cyclosporine A [2, 4, 5, 7]. However, 0.8% to 20% of the patients may present signs and symptoms of chronic LASIK-associated dry eye, persisting after 6 months [8, 9], in which case traditional treatment modalities are usually ineffective, thus requiring special management [2]. A better approach should aim to limit morbidity in these chronic cases by directing management towards the optimization of epithelial healing and possibly the neural mechanisms underlying LASIK-induced ocular surface changes and associated dry eye symptoms [10].

Blood derivatives such as autologous serum or a special preparation for topical eye application of platelet-rich plasma (E-PRP) have been shown to be powerful therapeutic tools in accelerating the healing response of the ocular surface and other different tissues [14–18]. This enhancing effect is attributed to growth factors and bioactive proteins that are synthesized and present in blood, especially in activated platelets' alpha-granules. Some articles published by our group have demonstrated the benefits of PRP in promoting optimal healing of dormant corneal ulcers of different etiologies and reducing symptoms and punctate keratopathy in moderate-to-severe DED and even neurotrophic keratopathy after LASIK [3, 17–22].

The present study aims to assess the efficacy of autologous platelet-rich plasma eye drop (E-PRP) monotherapy in the management of post-LASIK chronic OSS in a large prospective series of consecutive cases.

## 2. Materials and Methods

### 2.1. Study Design

This is a prospective interventional noncomparative consecutive clinical study. The ethical committee approval was obtained for the purpose of this prospective investigation. Adequate informed consent was signed by each one of the patients of the study. The investigation was carried out in accordance with the Declaration of Helsinki.

### 2.2. Patients

Adult patients suffering from moderate-to-severe dry eye syndrome for 6 months or more after conventional treatments with artificial tears following LASIK were included in the study. Surgical procedures were not standardized since up to 40% of the patients were referred from other services and therefore had undergone different methods of LASIK flap creation (i.e., mechanical microkeratome and femtosecond laser), different excimer laser platforms, and variable and often unknown amounts of ablated corneal stroma. Thus, this study did not intend to investigate the pathogenesis or assess the potential causes for the development of post-LASIK OSS but intended to evaluate the effectiveness of E-PRP monotherapy for the improvement of signs and symptoms in patients developing this condition.

Patients were classified according to the dry eye severity grading scheme proposed by the Dry Eye WorkShop (DEWS) [23], which takes into account the following parameters: ocular discomfort, visual symptoms, conjunctival injection, conjunctival and corneal fluorescein staining (CFS), corneal/tear signs, lid/meibomian glands, tear film break-up time (TBUT), and Schirmer II test score. All patients presented a tear break-up time (TBUT) between 4 and 9 seconds. Patients were treated with autologous PRP eye drops 6 times a day for 6 consecutive weeks. All previous forms of treatment were stopped 48 hours prior to receiving the autologous PRP.

Symptoms were evaluated based on the Ocular Surface Disease Index (OSDI) self-administered questionnaire, which was completed once at the time of enrollment in the study (baseline) and again after 6 weeks of treatment with E-PRP. Patients were also directly asked to report any change in their perception of ocular hyperemia, foreign body sensation, itching, photophobia, dryness, and pain.

Corrected distance visual acuity (CDVA) was measured in decimal notation with Snellen charts on a conventional projection screen, without using any eye drops for at least 1 hour before assessment. Conversion to logMAR units and lines of vision was posteriorly done to allow for assessment of visual improvement and statistical analysis.

Corneal fluorescein staining (CFS) was used to evaluate corneal superficial punctate epithelial keratitis. The modified Oxford scale, ranging from 0 (no staining dots) to 5 (worst staining condition), was used for this purpose. A decrease in CFS scores in at least one quadrant to total disappearance from baseline indicated improvement.

### 2.3. Autologous Platelet-Rich Plasma Preparation

The process to obtain PRP from autologous blood has been reported by our group previously [3]. PRP was prepared in optimal sterile conditions under a vented sterile fume hood. Blood drawn from patients was collected in 10 mL sterile tubes containing 1 mL sodium citrate acting as an anticoagulant. Enrichment of platelets in the plasma fraction (upper fraction) was achieved by the centrifugation of total blood at optimal conditions. Platelets were enriched by 2 to 2.5 times, according to quantitation performed through a coulter device. Under these conditions of centrifugation, leukocytes were dragged to the bottom of the tube and proteins and platelets remained in the upper fraction, enriching the plasma. Depending on the patient's hematocrit, the plasma fraction could vary from 25% to 50% of the total blood. Ninety percent of the plasma obtained after centrifugation was collected. Generally, 24 to 40 mL of platelet-rich plasma was obtained from 100 mL of the total blood. Mean values of blood cell count are shown in Table 1. The eye drop container was kept at 4°C for a maximum of 7 days, and the remaining vials were stored at −20°C for a maximum of 3 months.

### 2.4. The Main Outcome Measures

The main outcome measures were assessed at the end of the 6th week of uninterrupted treatment with E-PRP and included change in corrected distance visual acuity (CDVA), improvement of symptoms based on OSDI scores and/or patients' testimonial, decreased conjunctival hyperemia, and improvement in corneal fluorescein staining (CFS). All tests were performed at least 4 hours after the last topical instillation of PRP.

## 3. Results and Discussion

### 3.1. Results

One hundred and fifty-six eyes of 80 myopic patients were included in the study. Sixty-two patients were female (77.5%), and 18 patients were male (22.5%), with ages ranging from 22 to 82 years (mean 43.7 ± 12.3). All eyes underwent femtosecond laser-assisted myopic LASIK, with programmed spherical equivalent (SE) correction ranging from −2.00 to −7.50 diopters (D), being affected postoperatively by severe-to-moderate dry eye syndrome for 6 months or more.

The main outcomes are summarized in Table 2. Regarding symptoms after 6 weeks of uninterrupted treatment with E-PRP, 68 patients (85%) reported an improvement with respect to its previous condition, showing reduction or absence of symptoms, while 12 patients (15%) did not experience any change in symptoms. No patients reported worsening of symptoms.

**Figure 1:**
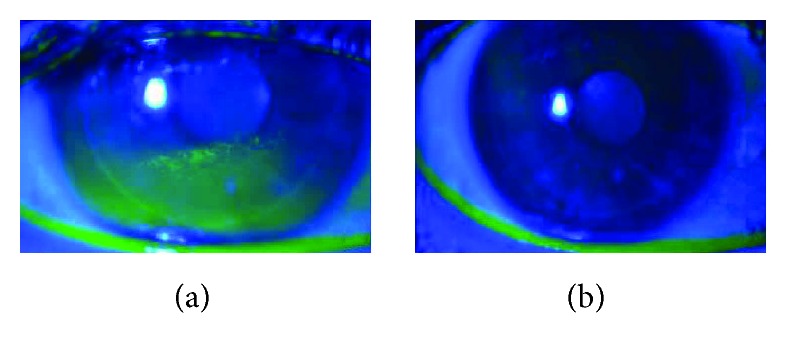
Patient with post-LASIK chronic OSS. (a) Before treatment, positive corneal fluorescein staining showing paracentral diffuse and coalescent areas of punctate keratitis in the inferior quadrant. (b) Transparent cornea, with complete resolution of previous punctate keratitis after treatment with E-PRP.

Positive corneal fluorescein staining (CFS) was present in 116 out of 156 eyes (74.3%) at baseline, with modified Oxford scale scores ranging from grades I to IV. Improvement indicated by a decrease of at least 1 point in the modified Oxford scale or improvement in at least one quadrant to total disappearance in CFS (as shown in Figure 1) was observed in 104 eyes (89.6%), while 10 eyes (8.6%) showed no change and 2 eyes (1.7%) presented slight worsening in superficial punctate keratitis. Three eyes (1.9%) presented severe punctate keratitis (grade IV) at baseline, all of which healed completely after 6 weeks of monotherapy with E-PRP.

Conjunctival hyperemia, present in 29 eyes (18.7%) at baseline, improved in 27 eyes (93.3%), while two eyes (6.7%) showed no change. No eyes had worsening in ocular surface inflammation, and none of the patients reported intolerance to the use of PRP eye drops, indicated by extreme discomfort or stinging sensation at the time of instillation.

**Figure 2:**
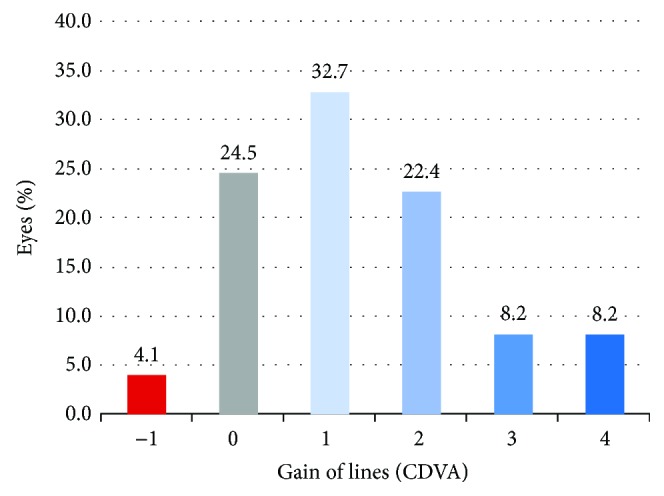
Visual change after treatment with autologous PRP eye drops in eyes presenting post-LASIK ocular surface syndrome and CDVA ≤ 0.9.

Mean decimal CDVA improved from 0.77 ± 0.25 (range 0.1 to 1.0) at baseline to 0.89 ± 0.17 (range 0.2 to 1.0) after treatment with PRP, which represents a statistically significant improvement in logMAR CDVA from 0.14 ± 0.19 to 0.06 ± 0.12 (*p* = 0.000). The number of eyes presenting decimal CDVA ≥ 0.8 increased from 101 (64.8%) to 124 (79.7%) before and after treatment, respectively. When considering eyes with decimal CDVA ≤ 0.9 before treatment (and therefore with some potential for visual improvement), 74 (71.4%) eyes improved at least 1 line of vision, 25 (24.5%) had no change, and 4 eyes (4.1%) lost 1 line of vision (Figure 2). All 53 eyes who had decimal CDVA = 1.0 at baseline maintained the same visual acuity. Visual improvement was significantly higher in eyes with worse CDVA at baseline (*p* = 0.000, *R* = −0.78) and was also directly correlated with improvement in the CFS score (*p* = 0.04, *R* = +0.60).

### 3.2. Discussion

Post-LASIK OSS is a complex, multifactorial, and nonuniform condition that affects patients in different levels of intensity and duration in the postoperative follow-up. There are many potential causes of tear dysfunction following LASIK, including corneal denervation, changes in corneal shape and tear distribution, and damage to goblet cells [4, 5]. Corneal denervation, secondary to LASIK flap creation and excimer laser stromal ablation, impairs the ocular surface-lacrimal gland functional unit, causing a spectrum of ocular surface conditions including reduced tear production, tear film instability, corneal and conjunctival epitheliopathy, symptoms of DED, and neuropathic pain states [2, 4, 5, 10].

While classification of the severity of DED in nonoperated eyes has already been shown to be quite difficult due to poor and even conflicting correlation between signs and symptoms in up to 40% of the patients [24, 25], this can be even more challenging in cases of post-LASIK OSS, mainly due to its primarily neuropathic etiology. For example, one patient with profound hypoesthesia and severe neuropathic epitheliopathy may not report any symptom of pain or discomfort, whereas another patient without any sign of dry eye (i.e., no CFS and normal TFBUT) may present intense neuropathic discomfort and even pain due to abnormal nerve fiber regeneration [26]. This apparent incompatibility between symptoms and objective findings has led to different definitions of post-LASIK “dry eye,” as well as varying methodologies in its assessment in both clinical practice and the literature, resulting in inconsistent results between published reports.

According to the 2007 International Dry Eye Workshop (DEWS), the prevalence of LASIK-induced dry eye in patients without dry eye history ranged between 0.25% and 48% [27]. Approximately half of all LASIK patients report dry eye symptoms up to 6 months after surgery, and tear film function and corneal sensitivity return to preoperative levels by 6 months after LASIK in most patients [1, 2, 10]. However, the occurrence of chronic OSS, indicated with a duration of at least 6 months, increases by 18–41% after LASIK [10] and has been reported in 0.8% to 20% of the patients [8, 9].

LASIK-induced neurotrophic epitheliopathy (LINE) is seen in about 4–14% of the LASIK patients. It usually reaches a severity peak between the first week and the third month after LASIK and then returns to preoperative levels by the sixth month [4, 5, 10]. Corneal nerve damage caused by LASIK has been shown to be persistent, with corneal nerve fiber density between 6 months and 2 years following LASIK being significantly lower than preoperative levels. Altered tear concentrations of nerve growth factor (NGF) and other neuropeptides, such as substance P (SP) and calcitonin gene-related peptide (CGRP), may also be key factors in LINE's pathophysiology [10]. Altered corneal nerve morphology parameters—lower nerve density, thinner width, fewer interconnections, and higher tortuosity—have been shown by in vivo laser scanning confocal microscopy at 6 to 12 months after LASIK, along with higher dry eye symptoms, CGRP levels, and conjunctival sensitivity with respect to nonoperated healthy eyes [28].

LASIK has also been associated with loss of goblet cells due to the pressure applied over the perilimbal conjunctiva by the suction ring to create the flap either with femtosecond laser or with microkeratome [13], which leads to decreased production of goblet cell mucin and, hence, tear film instability. It may take up to 6 months for goblet cell density to return to baseline values after LASIK [13]. Studies suggest that tear film break-up time (BUT) is decreased after LASIK [29–31] and Schirmer II scores range from no change to moderate decrease [8, 32].

Bower et al. [8] found a significant decrease in TBUT and an increase in rose bengal corneal staining up until 12 months after LASIK when compared to preoperative findings but did not find a significant decrease in Schirmer II test at any time point along the follow-up, suggesting that chronic tear film changes after LASIK are rather qualitative, such as decreased levels of goblet cell mucin and neurotrophic factors, reduced blink rate, and changes in corneal shape and tear film distribution. Therefore, patients who develop post-LASIK chronic OSS should be better treated with therapeutic tools that not only promote ocular surface lubrication but also produce trophic changes for an effective and sustained healing process.

Autologous PRP is a preservative-free biological product from the patient's own blood, and its effectiveness is determined by the presence of a high concentration of platelets, whose alpha-granules contain storage pools of growth factors, including platelet-derived growth factors, transforming growth factor *β* (TGF-*β*), epithelial growth factors, fibroblast growth factors, insulin-like growth factor I, and vascular endothelial growth factors, as well as cytokines (such as PF4 and CD40L), chemokines, and newly synthesized active metabolites [19]. These products are involved in proliferation, migration, and differentiation of corneal epithelial cells, thus helping to heal and maintain the proper condition of the ocular surface. Autologous PRP has been used successfully in other ocular surface disorders such as moderate-to-severe DED (both evaporative and aqueous deficiency), persistent epithelial defects, alkali burn, dormant ulcers, and corneal surface reconstructions in corneal perforations [3, 15–22]. E-PRP can be stored at −20°C for up to 3 months maintaining constant or slight variations in the concentration of the most important growth factors.

In a prospective controlled randomized study by our group [21], we have found that the use of E-PRP eye drops for standard postoperative treatment following LASIK had beneficial effects for promoting epithelial healing after LASIK but had no objective “extra” positive effect on the recovery of corneal sensitivity or regeneration of the corneal subbasal nerve layer as shown in confocal microscopy. Although positive results have been demonstrated in both experimental and clinical investigations regarding the speed of the peripheral nerve regeneration when using PRP for more severe epitheliopathies such as neurotrophic ulcers, we believe the same effect was not obtained in our previous study due to the limited bioavailability of growth factors in corneal stroma when the substance is topically administered [21]. Growth factors present in PRP are large molecules (i.e., 30,000 kilodaltons for platelet-derived growth factor) and are composed in part of hydrophilic units, which limit its penetration across the fully or relatively intact corneal epithelium after LASIK. The epithelial status after LASIK is much distinct from that in other more severe epitheliopathies such as neurotrophic ulcers, in which a lack of epithelial integrity would facilitate the penetration and action of PRP on the damaged nerves. Indeed, in another study conducted by our group to assess the effects of autologous PRP in the treatment of dormant corneal ulcers [19], we have shown that topical E-PRP promoted complete healing in 13 cases (50%), while 11 (42%) improved significantly and only 2 (8%) showed no change in biomicroscopy examination. Moreover, PRP has been shown to reduce pain, inflammation, and photophobia and to improve vision in the majority of the patients with dormant corneal ulcers included in that study [19].

We have found that 15% of the patients did not show improvement in symptoms after treatment. Nettune and Pflugfelder [2] have advocated that corneal nerves that regenerate 6 months to 2 years after LASIK surgery are different from preexisting nerves, and this abnormal nerve regeneration may be responsible for much of the irritation sensation reported after LASIK. This neurally mediated spectrum of post-LASIK OSS may vary from mild but persistent sensation characteristic of tear dysfunction to severe and disabling chronic pain states. Confocal microscopy suggests that these patients may have abnormal regeneration of the subbasal nerve plexus. Thus, ocular surface symptoms experienced by some after LASIK may be misattributed to tear dysfunction, when in fact they are due to the altered sensitivity and ectopic firing of peripheral nerves. Tuisku et al. found that even 2 to 5 years after LASIK for high myopia, most patients experienced ocular surface discomfort consistent with dry eye syndrome despite the absence of clinical signs of ocular surface disease and with normal sensitivity when measured with noncontact esthesiometry [23]. This would explain why some patients in our study did not show improvements in symptoms despite a better status of the ocular surface after treatment with E-PRP.

In the present study, we show evidence that supports the beneficial effects of monotherapy with autologous E-PRP on patients with post-LASIK chronic OSS. Of the 113 (74.3%) eyes presenting chronic punctate keratitis 6 months after LASIK (baseline), 101 (89.4%) showed significant improvement after treatment with E-PRP. There was a significant improvement in CDVA, which correlated positively with the recovery of the epithelial status, and 71.4% of the eyes improved at least 1 line in CDVA. E-PRP had a remarkable effect on the relief of symptoms of DES and improvement of conjunctival hyperemia and inflammation. Autologous PRP was well tolerated, and no adverse effects were observed during the clinical study.

## 4. Conclusions

In conclusion, monotherapy with autologous PRP eye drops has been shown to be a very good option for the treatment of post-LASIK chronic OSS. Significant advantages are ease of preparation, absence of preservatives, its autologous origin, safety, and minimal or no intolerance, with no observed side effects.

## Figures and Tables

**Table 1:** Blood cell count before (whole blood) and after centrifugation (PRP).

	Platelets × 10^3^/*μ*L	RBC × 10^6^/*μ*L	WBC × 10^3^/*μ*L
Whole blood (mean ± SD)	193.77 ± 51.86	4.12 ± 0.40	4.91 ± 1.92
PRP (mean ± SD)	342.07 ± 89.04	0.03 ± 0.04	1.09 ± 0.75
Concentration index (mean)	1.85	—	—
Reduction (%)	—	−99.3	−77.8

*μ*L: microliter; RBC: red blood cells; WBC: white blood cells; SD: standard deviation; PRP: platelet-rich plasma.

**Table 2:** Signs and symptoms of post-LASIK ocular surface syndrome before and after monotherapy with autologous platelet-rich plasma eye drops.

	Number of cases before treatment *n* (%)	Worsening	No change	Improvement
Dry eye symptoms (patients)	80 (100%)	0 (0%)	12 (15%)	68 (85%)
Fluorescein staining (eyes)	116 (74.3%)	2 (1.7%)	10 (8.6%)	104 (89.6%)
Conjunctival hyperemia (eyes)	29 (18.7%)	0 (0%)	2 (6.7%)	27 (93.3%)
